# Supplementary material for: Heterogeneous generation of new cells in the adult echinoderm nervous system
Source: Front Neuroanat. 2015 Sep 22;9:123. doi: 10.3389/fnana.2015.00123 (PMC4585025; doi:10.3389/fnana.2015.00123)
Supplement: Additional File 2 — R code used to perform statistical computations. [file DataSheet2.PDF]

```
# This file contains sample R code that can be used to reproduce
our calculations.
# The lines preceded with the pound sign symbol (#) are comments
or sample output lines.

#Reading the data file (assuming its name is "input.csv"):
data <- read.csv("input.csv", header = T)

#The L_R_Position column is currently interpreted as "integer",
#it should be converted to "factor" to ensure that it is treated
as a categorical variable:
data$L_R_Position <- as.factor(data$L_R_Position)
class(data$L_R_Position)
 #[1] "factor"
#Exluding empty rows, i.e., where BrdU._vs_area is NA:
data <- subset(data, !is.na(BrdU._vs_area))

#====================#
#== NORMALITY TEST ==#
#====================#
#fitting the model:
res <- residuals(lm(BrdU._vs_area ~
TimePoint*A_B_position*L_R_Position, data))

#Drawing a histogram:
hist(res)

#Running the Shapiro-Wilk test for normality:
shapiro.test(res)

# Shapiro-Wilk normality test
#
# data:  res
# W = 0.8747, p-value = 2.478e-10

#==========================#
#== ANALYSIS OF DEVIANCE ==#
#==========================#

#Since the data are non-normal, we employ analysis of deviance
for generalized linear model fits:

glm <- glm(BrdU._vs_area ~ TimePoint*A_B_position*L_R_Position,
family = quasipoisson, data)
anodev <- anova(glm, test = "F")
```

```
anodev
# Analysis of Deviance Table
#
# Model: quasipoisson, link: log
#
# Response: BrdU._vs_area
#
# Terms added sequentially (first to last)
#
#
#                                       Df  Deviance Resid. Df
Resid. Dev       F    Pr(>F)
#   NULL                                                   159
0.108670
#   TimePoint                            3 0.0072539       156
0.101417  6.5561 0.0003836 ***
#   A_B_position                         1 0.0047194       155
0.096697 12.7962 0.0005019 ***
#   L_R_Position                         4 0.0263329       151
0.070364 17.8499 1.597e-11 ***
#   TimePoint:A_B_position               3 0.0122447       148
0.058120 11.0669 1.819e-06 ***
#   TimePoint:L_R_Position              12 0.0062077       136
0.051912  1.4027 0.1738366
#   A_B_position:L_R_Position            4 0.0035387       132
0.048373  2.3987 0.0538735 .
#   TimePoint:A_B_position:L_R_Position 12 0.0026968       120
0.045676  0.6093 0.8308328
#   ---
#   Signif. codes:  0  0.001  0.01  0.05  0.1  1

# Analyzing further the main effect of the position along the
left-right axis
#Performing pairwise comparisons for the "L_R_Position"
categorical variable:
library(multcomp)
pairwise_L_R_Position <- glht(glm, mcp(L_R_Position = "Tukey"))
summary(pairwise_L_R_Position, test = adjusted("fdr"))

# Simultaneous Tests for General Linear Hypotheses
#
# Multiple Comparisons of Means: Tukey Contrasts
#
#
# Fit: glm(formula = BrdU._vs_area ~ TimePoint * A_B_position *
```

```
L_R_Position,
#           family = quasipoisson, data = data)
#
# Linear Hypotheses:
#              Estimate Std. Error z value Pr(>|z|)
# 2 - 1 == 0 -1.818522   0.648267  -2.805   0.0170 *
# 3 - 1 == 0 -2.953067   1.087682  -2.715   0.0170 *
# 4 - 1 == 0 -0.564919   0.402359  -1.404   0.2097
# 5 - 1 == 0 -0.008949   0.343323  -0.026   0.9792
# 3 - 2 == 0 -1.134544   1.218999  -0.931   0.3911
# 4 - 2 == 0  1.253603   0.681762   1.839   0.1099
# 5 - 2 == 0  1.809574   0.648674   2.790   0.0170 *
# 4 - 3 == 0  2.388147   1.107972   2.155   0.0623 .
# 5 - 3 == 0  2.944118   1.087925   2.706   0.0170 *
# 5 - 4 == 0  0.555971   0.403014   1.380   0.2097
# ---
#   Signif. codes:  0  0.001  0.01  0.05  0.1  1
# (Adjusted p values reported -- fdr method)

#Producing a boxplot of BrdU+ cell density along the L_R-axis
regardless of other factors:
boxplot(BrdU._vs_area ~ L_R_Position, data, notch = T)

#=================================================================
=========================

# Since there is a significant interation between the post
injection time and apical vs basal position, we divide the
original
#dataset into two parts, to study the effect of time separately
in the apical and basal regions of the RNC:

data_apical <- subset(data, A_B_position == "apical")
data_basal <- subset(data,  A_B_position == "basal")

#With datasets representing the apical and basal regions of the
RNC, we now run analysis of deviance for each of them to
investigate
#the impact of time on density of BrdU-labeled cells:

###########
# Apical: #
###########
glm_apical <- glm(BrdU._vs_area ~ TimePoint, family =
quasipoisson, data_apical)
```

```
anodev_apical <- anova(glm_apical, test = "F")
anodev_apical

# Analysis of Deviance Table
#
# Model: quasipoisson, link: log
#
# Response: BrdU._vs_area
#
# Terms added sequentially (first to last)
#
#
#            Df  Deviance Resid. Df Resid. Dev      F Pr(>F)
# NULL                          79   0.055036
# TimePoint  3 0.0028659        76   0.052171 1.4364 0.2388

#########
# Basal:#
#########
glm_basal <- glm(BrdU._vs_area ~ TimePoint, family =
quasipoisson, data_basal)
anodev_basal <- anova(glm_basal, test = "F")
anodev_basal
# Analysis of Deviance Table
#
# Model: quasipoisson, link: log
#
# Response: BrdU._vs_area
#
# Terms added sequentially (first to last)
#
#
#            Df Deviance Resid. Df Resid. Dev      F    Pr(>F)
# NULL                         79   0.048915
# TimePoint  3 0.016633        76   0.032282 10.729 5.864e-06 ***
#   ---
#   Signif. codes:  0  0.001  0.01  0.05  0.1  1

#Multiple comparisons for TimePoint in the apical region of the
RNC:
pairwise_basal_time <- glht(glm_basal, mcp(TimePoint = "Tukey"))
summary(pairwise_basal_time, test = adjusted("fdr"))

# Simultaneous Tests for General Linear Hypotheses
#
```

```
# Multiple Comparisons of Means: Tukey Contrasts
#
#
# Fit: glm(formula = BrdU._vs_area ~ TimePoint, family =
quasipoisson,
#          data = data_basal)
#
# Linear Hypotheses:
#                       Estimate Std. Error z value Pr(>|z|)
# 1week - 0weeks == 0    1.2328     0.4229   2.915  0.00711 **
# 5weeks - 0weeks == 0   0.9790     0.4365   2.243  0.02989 *
# 8weeks - 0weeks == 0   1.8360     0.4007   4.582 2.77e-05 ***
# 5weeks - 1week == 0   -0.2539     0.3040  -0.835  0.40362
# 8weeks - 1week == 0    0.6031     0.2499   2.413  0.02371 *
# 8weeks - 5weeks == 0   0.8570     0.2722   3.148  0.00493 **
#   ---
#   Signif. codes:  0  0.001  0.01  0.05  0.1  1
# (Adjusted p values reported -- fdr method)

# Producing a boxplot showing changes in density of BrdU-labeled
cells in time in the apical and basal
#regions of the RNC:
boxplot(BrdU._vs_area ~ TimePoint, data_apical, outline = F)

boxplot(BrdU._vs_area ~ TimePoint, data_basal, outline = F)

#================================================================
==========================

#Now we are going to compare the density of BrdU-labeled cells
between the apical and basal
#regions at 0 weeks and at 8 weeks

data_0weeks <- subset(data, TimePoint == "0weeks")
data_8weeks <- subset(data, TimePoint == "8weeks")

#Apical vs basal, 0 weeks
glm_0weeks <- glm(BrdU._vs_area ~ A_B_position, family =
quasipoisson, data_0weeks)
anodev_0weeks <- anova(glm_0weeks, test = "F")
anodev_0weeks

# Analysis of Deviance Table
#
# Model: quasipoisson, link: log
```

```
#
# Response: BrdU._vs_area
#
# Terms added sequentially (first to last)
#
#
#               Df  Deviance Resid. Df Resid. Dev      F
Pr(>F)
# NULL                            39   0.028556
# A_B_position  1 0.0096305        38   0.018925 22.561 2.891e-05
***
#   ---
#   Signif. codes:  0  0.001  0.01  0.05  0.1  1

boxplot(BrdU._vs_area ~ A_B_position, data_0weeks, outline = F)

#Apical vs basal, 8 weeks:
glm_8weeks <- glm(BrdU._vs_area ~ A_B_position, family =
quasipoisson, data_8weeks)
anodev_8weeks <- anova(glm_8weeks, test = "F")
anodev_8weeks
# Analysis of Deviance Table
#
# Model: quasipoisson, link: log
#
# Response: BrdU._vs_area
#
# Terms added sequentially (first to last)
#
#
#               Df  Deviance Resid. Df Resid. Dev      F Pr(>F)
# NULL                            39   0.031664
# A_B_position  1 0.0017656        38   0.029899 1.8326 0.1838

boxplot(BrdU._vs_area ~ A_B_position, data_8weeks, outline = F)
```
