# Supplementary material for: Heterogeneous generation of new cells in the adult echinoderm nervous system
Source: Front Neuroanat. 2015 Sep 22;9:123. doi: 10.3389/fnana.2015.00123 (PMC4585025; doi:10.3389/fnana.2015.00123)
Supplement: Additional File 3 — Analysis of deviance table showing the effects of the length of post-injection time, position along the apical-basal axis and left-right axis on the density of BrdU-labeled cells in the ectoneural neuroepithelium of the radial nerve cord of the sea cucumber H. glaberrima. [file DataSheet3.PDF]

Analysis of Deviance Table

Model: quasipoisson, link: log

Response: Density_of_BrdU-labeled_cells

Terms added sequentially (first to last)

| | Df | Deviance | Resid. Df | Resid. Dev | F | Pr(>F) | |
|---|---|---|---|---|---|---|---|
| NULL | | | 159 | 0.108670 | | | |
| TimePoint | 3 | 0.0072539 | 156 | 0.101417 | 6.5561 | 0.0003836 | *** |
| A_B_position | 1 | 0.0047194 | 155 | 0.096697 | 12.7962 | 0.0005019 | *** |
| L_R_Position | 4 | 0.0263329 | 151 | 0.070364 | 17.8499 | 1.597e-11 | *** |
| TimePoint:A_B_position | 3 | 0.0122447 | 148 | 0.058120 | 11.0669 | 1.819e-06 | *** |
| TimePoint:L_R_Position | 12 | 0.0062077 | 136 | 0.051912 | 1.4027 | 0.1738366 | |
| A_B_position:L_R_Position | 4 | 0.0035387 | 132 | 0.048373 | 2.3987 | 0.0538735 | . |
| TimePoint:A_B_position:L_R_Position | 12 | 0.0026968 | 120 | 0.045676 | 0.6093 | 0.8308328 | |

---

Signif. codes: 0 ‘***’ 0.001 ‘**’ 0.01 ‘*’ 0.05 ‘.’ 0.1 ‘ ’ 1

where

TimePoint - Length of time after the last BrdU injection

A_B_position - position along the apical-basal axis

L_R_Position - position along the left-right axis
