# Supplementary material for: Heterogeneous generation of new cells in the adult echinoderm nervous system
Source: Front Neuroanat. 2015 Sep 22;9:123. doi: 10.3389/fnana.2015.00123 (PMC4585025; doi:10.3389/fnana.2015.00123)
Supplement: Additional File 4 — Reference numbers of sequences used to generate phylogenetic trees. [file DataSheet4.PDF]

###############
Churchill
###############
BC105418
BC019472
AF238863
AF238862
NM_001141582
BC085457
NM_001204691
NM_001168045
FN357283

##########
ELAV
##########
L26405
U29148
CR761704
BC135736
D31953
S83320
CR761432
CR859234
U29088
BC074585
U38175
AK080365
XM_003729624
GQ223115
NM_001164904
M21152
M61748
AF026145
X98370

##########
DCLK
##########
NM_033403
NM_172928
XM_001236016
NM_053343
NM_004734
NM_019978
XM_005172671
BC168500
XM_420439
NM_001040260
NM_027539
XM_006232679
NM_001168056
XM_787378
NM_000555
AF155959
NM_010025
NM_067170
BC045136
AF181721

NM_146246
NM_178857
XM_426089
NM_011283
NM_173958
AF143222

###########
FoxJ1
###########
BC046460
BC082543
AJ609390
AJ609391
EU599363
EU581680
GU224261
NM_001079545
NM_021899
NM_001005675
NM_001078246
GU224261
DQ286737
NM_001110046
BC151828
NM_172699
X60787
EU882160
L26507
NM_005250
NM_008024
AK077935
AY204207
NM_001086898
BC029778
NM_023907

#########
Hes
#########
U36194
NM_001085917
BC059551
BC039152
BC018375
NM_131873
AF032966
AY349472
AY349473
AY349467
AY349471
AY349468
AY349469
NM_001164994
SPU_006814
GU251976
SPU_021608
AB035178
AB035179

NM_019089
BC138112
D14029
DQ272660
D32132
D12516
SPU_009465
AJ272215
AF172288
AF151521
AF151522
AJ249545
AF172287
JN982707

############
Msi1/2
############
NM_002442
NM_008629
BC090916
BC017560
BC111809
NM_168718
NM_001164892
AY313138
NM_001088619
AF181719
AF225910
M65028
BC043069
NM_001011015
NM_001031142
BC021374

############
Lhx1/5
############
F291181
L35572
L42547
L37802
L35569
U14755
AB049118
EU307289
BC040321
AF226662
BC119169
L39880
AF156888
Z22702
U34590
AK034177
AF353304
BC095186
L35567
S69329
D21135

AK001022
AK013964
U11701
AF124734
BC080067
L35566
AB188254

############
NeuroD
############
U50822
D82075
Y09597
U28067
D85188
AF203901
D85845
AF115774
U58681
U58471
AF115772
AF063609
D44480
BC123740
AJ133776
Y09167
BC036847
U76207
U63842
U63841
L36646

#########
NFI
#########
NM_205272
BC014290
BC120107
NM_001097167
NM_001095703
BX004774
NM_205271
BC012120
U57635
NM_001127983
X61225
CR361565
BC022264
BC075702
NM_012988
NM_205870
XM_003727226
XM_002119942

##########
Piwi
##########
AF104260

AF438405
NM_001098852
NM_183338
XM_004063189
AB079368
AB258534
NM_152431
JX036538
NM_214600
AY493987
EU817487
AB079367
AF285586
AB455103
AF104354
DQ186985
NM_179453
AJ223508
NM_079010
NM_153403

##########
Prox
##########
U44060
AF061576
U46563
NM_131405
NM_001192679
NM_175198
NM_001191771
NM_001168164
JQ956375
M81389
AJ571696
NM_066359
JQ425149

##########
Runt
##########
NM_204128
D14636
NM_212858
Z35278
AF155880
AF035446
L34598
L35271
AY146615
X56432
DQ334859
AF217651
NM_001164957
EU424344
NM_214614
AF051770
AB027412
JF720854

EU877198
NM_001280849
