# Supplementary material for: Heterogeneous generation of new cells in the adult echinoderm nervous system
Source: Front Neuroanat. 2015 Sep 22;9:123. doi: 10.3389/fnana.2015.00123 (PMC4585025; doi:10.3389/fnana.2015.00123)
Supplement: Additional File 5 — Sequences of PCR primers used to generate templates for riboprobe synthesis. [file DataSheet5.PDF]

Gene	Primer	Sequence (5'- 3')
Churchill	Chch_F2	CCTCTGCCACACAAAATACAAAG
	Chch_R2	CTACATTGGATTTGAGCCTTACC
DCLK	DCLK_F2	GATGGCAGTCCTTTTCGTTG
	DCLK_R2	GCTGGATTCTGGGATGGTT
ELAV	ELAV_F3	TGATGGCTTGTCTGGTGGT
	ELAV_R3	TCCTTTACGTTTATTTATGTTCACA
FoxJ1	Foxj1_F1	TCCATATCAGTGTGGCATAAGAGT
	Foxj1_R1	TTGGTTGGCACAGAATCAGC
Hes	Hes_F1	GAAAAGAGGCGACGAGCAA
	Hes_R1	TCAAGTCAACCAAGTTAGAATCCA
Klf1/2/4	Klf_F2	ATTCACCACCTCCCCAGTTC
	Klf_R2	ACTTCCAGCCACAGCCCTT
Lhx1/5	Lhx_F1	AGAGTCCGTATTAGTTCCGTTCA
	Lhx_R1	CCACCAGGTCACCATCATCC
Msi1/2	Msi_F2	CCACAAGAAGAAGTAGAGGGTTTG
	Msi_R2	AAGCCTGCCTGAAATGGACT
Myc	Myc_F5	GCTCTACAGAAAAGGGAACTTGA
	Myc_R5	CAGTTTCATTATTTCCCGAGTGTC
NeuroD	NeuroD_F2	GAAGGTTTCCAGGATAGTGTTG
	NeuroD_R2	GCATACGGTTTCTTTCACGG
NFI	NFI_F3	GCCAAGTCTAAGTGAAGCAACATC
	NFI_R3	AAATTGGTGGGACTGGTATGAAGT
Oct1/2/11	Oct_F1	TGAAAGTATCGCCAGAAGACG
	Oct_R1	AATGGAGACCTGACGGAGAAC
Prox	Prox_F1	GGGTGATTCGGCAGACTTG
	Prox_R1	AAGGGGTGGCATAAAGGTTCT
Piwi	Piwi_F7	GTGTCGTGCCTGGACAAAGA
	Piwi_R7	ATCATATCGTTCTTTCCTTGCG
Runt	Runt_F4	TGGATTTGGAAGACCGTACC
	Runt_R4	TCTAAACCGCCCATTTGATG
SoxB1	SoxB1_F2	GTCGTTCGCAACATCACCT
	SoxB1_R2	ACTTCACATCCAGCATACCTCA
GFP	EGFP_F1	CCACAAGTTCAGCGTGTCC
(control)	EGFP_R1	TGCTCAGGTAGTGGTTGTCG
