# Supplementary figures and images for: Heterogeneous generation of new cells in the adult echinoderm nervous system
Source: Front Neuroanat. 2015 Sep 22;9:123. doi: 10.3389/fnana.2015.00123 (PMC4585025; doi:10.3389/fnana.2015.00123)

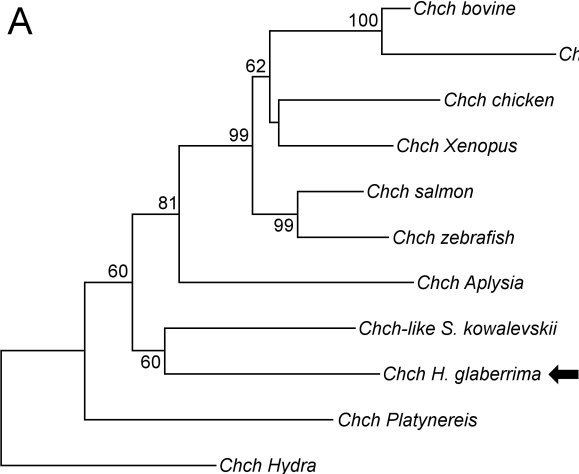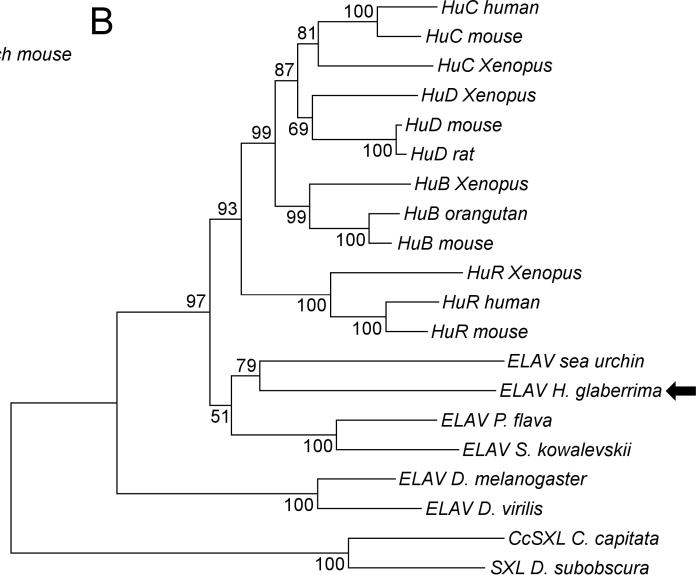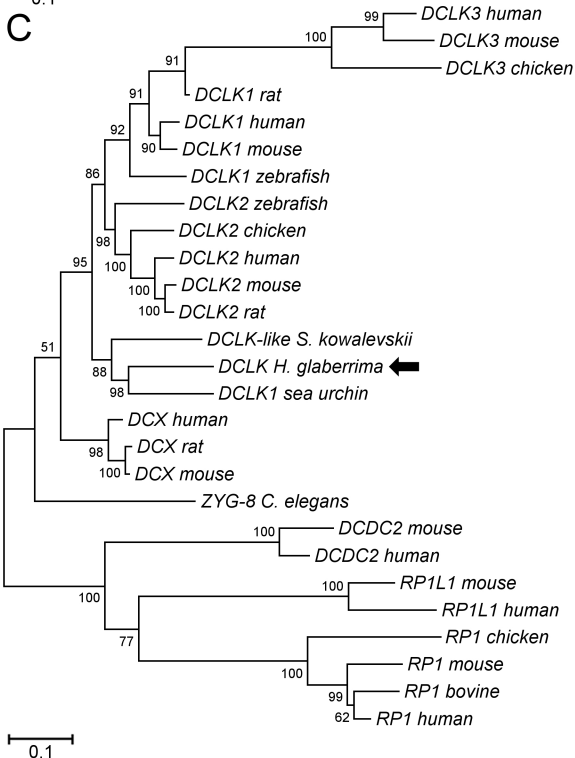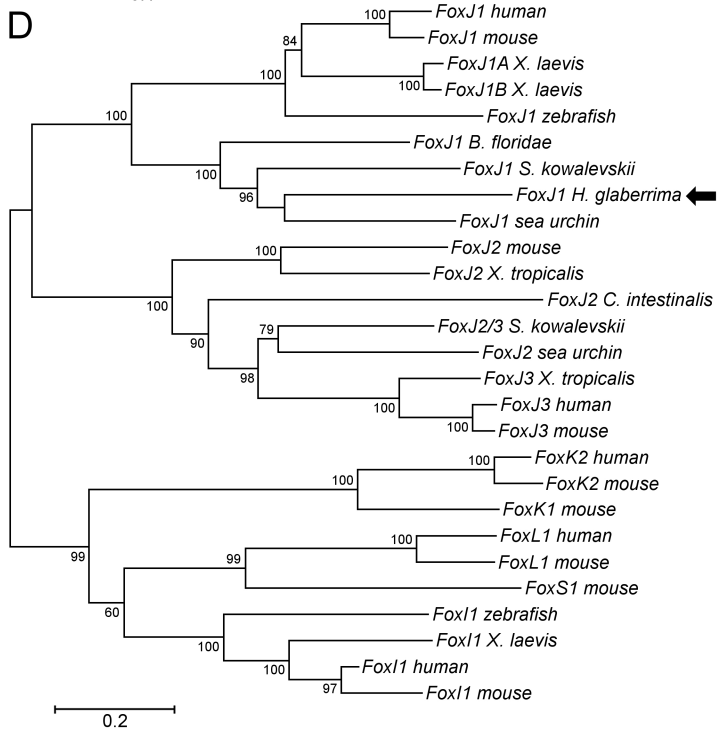

Supplement: Additional File 6 — Neighbor-joining trees showing phylogenetic relationships of H. glaberrima Churchill (A), ELAV (B), DCLK (C), and FoxJ1 (D) (arrows) with homologous genes from other organisms. Bootsrap values higher than 50% (2000 replicates) are shown next to the branches. [file DataSheet6.PDF]

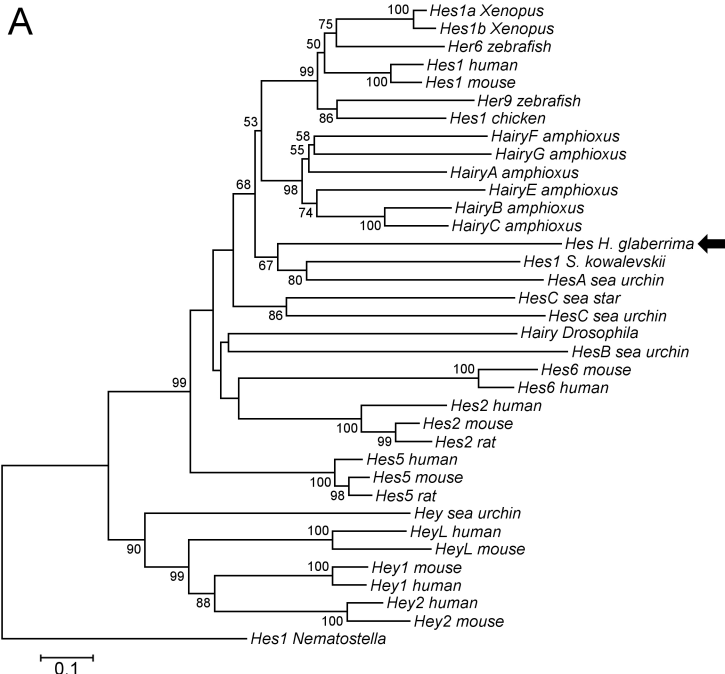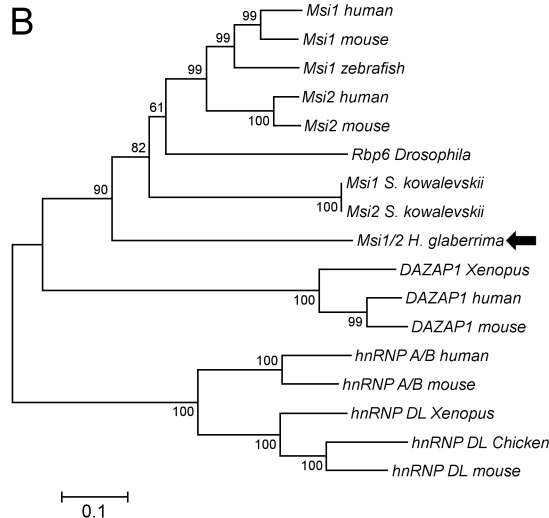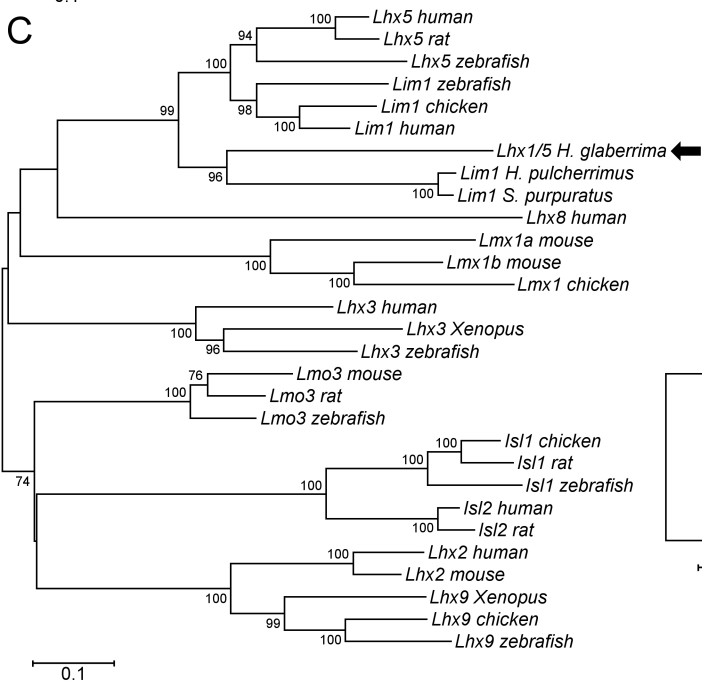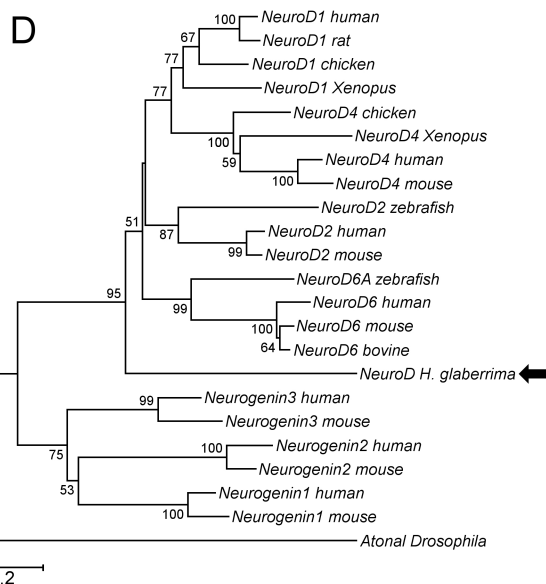

Supplement: Additional File 7 — Neighbor-joining trees showing phylogenetic relationships of H. glaberrima Hes (A), Msi1/2 (B), Lhx1/5 (C), and NeuroD (D) (arrows) with homologous genes from other organisms. Bootsrap values higher than 50% (2000 replicates) are shown next to the branches. [file DataSheet7.PDF]

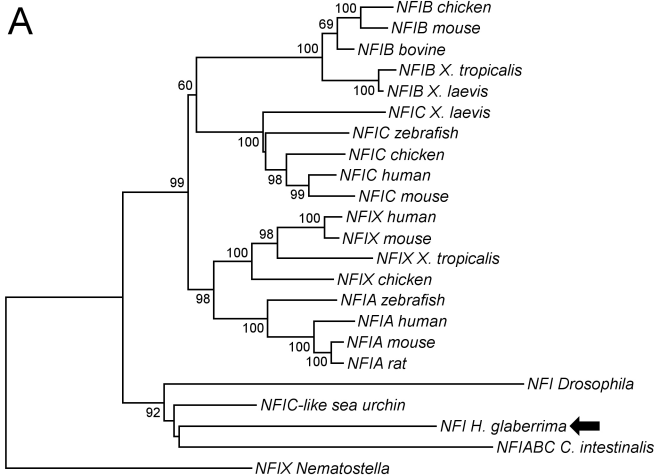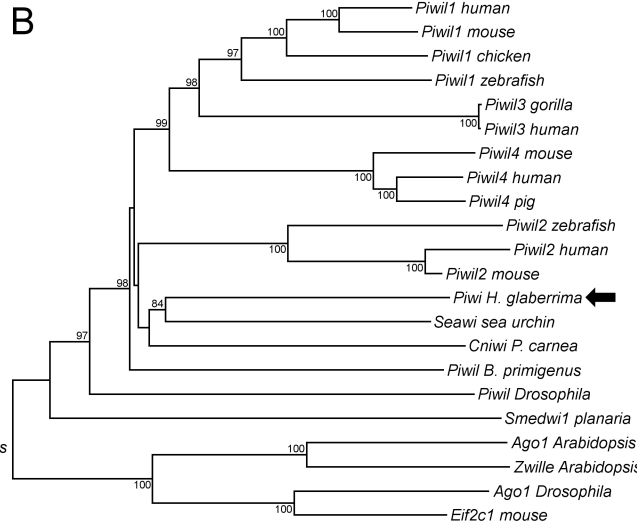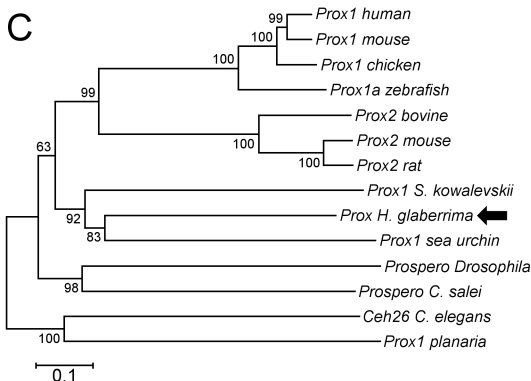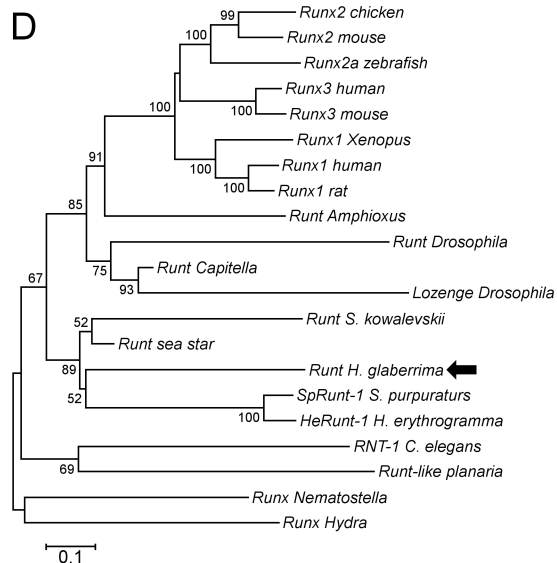

Supplement: Additional File 8 — Neighbor-joining trees showing phylogenetic relationships of H. glaberrima NFI (A), Piwi (B), Prox (C), and Runt (D) (arrows) with homologous genes from other organisms. Bootsrap values higher than 50% (2000 replicates) are shown next to the branches. [file DataSheet8.PDF]
